# Integrated Transcriptome and Proteome Analysis Provides New Insights into Starch and Sucrose Metabolism and Regulation of Corm Expansion Process in *Colocasia esculenta*

**DOI:** 10.3390/biology14020173

**Published:** 2025-02-08

**Authors:** Chengwu Zou, Fanglian He, Huinan Li, Lili Liu, Zuyang Qiu, Weiqing Dong

**Affiliations:** 1 State Key Laboratory for Conservation and Utilization of Subtropical Agro-Bioresources, College of Agriculture, Guangxi University, Nanning 530004, China; zouchengwu@gxu.edu.cn; 2 Vegetable Research Institute, Guangxi Zhuang Autonomous Region Academy of Agricultural Sciences, Nanning 530007, China; hefanglian@gxaas.net (F.H.);; 3 Lipu City Agricultural and Rural Bureau, Lipu 546600, China

**Keywords:** *Colocasia esculenta*, corm expansion, full-length transcriptome, proteome, metabolism regulation

## Abstract

The global significance of *Colocasia esculenta*, a tuber crop rich in nutritional value and starch, prompts further investigation into its corm development. Background: Previous studies have focused on starch accumulation within the tubers, yet the genetic and proteomic basis of corm expansion remains largely unexplored. This study aims to elucidate the key genes and proteins involved in this process. Methods: We selected ‘Lipu Taro No.1’ and conducted a longitudinal starch content analysis, full-length transcriptome sequencing, and a proteomic analysis during three distinct stages of corm development. Results: Our findings reveal a significant increase in both amylose and amylopectin contents as the corm develops, indicating the temporal regulation of starch biosynthesis. The integration of transcriptome and proteomic data identified differentially expressed genes and proteins associated with starch and sucrose metabolism, as well as plant hormone signal transduction. Conclusions: This study delineates a temporal gene expression pattern that is crucial for starch synthesis and provides insights into the regulatory mechanisms controlling corm expansion and starch deposition, offering valuable references for future molecular breeding strategies to enhance taro yield and quality.

## Simple Summary

This study focuses on *Colocasia esculenta*, a globally significant tuber crop known for its nutritional value and starch content. While previous research has explored starch accumulation in tubers, the genes and proteins involved in corm expansion are not well understood. This research investigates the ‘Lipu Taro No.1’ variety, analyzing its starch content and conducting full-length transcriptome sequencing and proteomic analysis at the different stages of corm development. Findings show that both amylose and amylopectin contents increase with corm development, indicating temporal regulation of starch biosynthesis. By integrating transcriptome and proteomic data, this study identifies differentially expressed genes and proteins related to starch and sucrose metabolism and plant hormone signal transduction. This reveals a link between starch content changes and gene and protein expression in taro corms. This study provides insights into the temporal gene expression pattern for starch synthesis during corm development, offering valuable information for understanding the regulatory mechanisms of corm expansion and starch deposition, and for developing molecular breeding strategies to improve taro yield and quality.

## 1. Introduction

*Colocasia esculenta* (L.) Schott, a member of the Araceae family, holds a historically significant position as a globally cultivated crop, predominantly in tropical and subtropical regions. This crop serves as a crucial food source for approximately 500 million people across Asia, Africa, Central America, and the Pacific Islands [1]. In particular, China boasts a rich history of taro cultivation spanning over 2000 years [2,3]. The taro corm, which functions both as a primary organ for nutrient storage and asexual reproduction, also holds substantial economic importance. According to recent statistics, China’s taro production reached 1.912 million tons in 2021, accounting for 15.43% of the global total and ranking third worldwide. In the same year, China exported 64,900 tons of taro, valued at USD 73.578 million, securing the top position globally in terms of export value [4].

As a starchy root crop, the taro corm constitutes the main edible portion, with starch comprising 70% to 80% of its dry matter. This high starch content plays a pivotal role in determining the yield and quality of taro [5]. Additionally, taro corms are rich in carbohydrates, fats, crude fiber, vitamin C, B vitamins, high-quality protein, and essential minerals such as calcium, potassium, and phosphorus, contributing to their overall nutritional value. Notably, taro boasts twice the carbohydrate content of potatoes and an 11% higher protein content compared to yams, cassava, and sweet potatoes [6]. Furthermore, taro’s moderate Glycemic Index (GI) positions it as an ideal dietary carbohydrate alternative for diabetic patients [1,7].

Among the various taro cultivars, Lipu Taro No. 1, bred by the Guangxi Academy of Agricultural Sciences, stands out for its exceptional yield and superior culinary quality. This variety exhibits a growth cycle ranging from 210 to 240 days and possesses distinctive morphological traits, including the absence of stolons, a spindle-shaped mother corm, individual corm weights between 1.5 and 3 kg, and an impressive yield per hectare of 33,000 to 42,000 kg (unpublished data). Lipu Taro No. 1 is renowned for its rich aroma and fine, waxy texture, distinguishing it as one of the most aromatic and finely textured varieties among taro cultivars. The intensity of its aroma and the fineness of its texture are vital indicators for assessing taro’s culinary quality, and Lipu Taro No. 1 excels in both aspects. However, despite these notable attributes, the underlying regulatory mechanisms governing corm enlargement and quality formation in this variety remain incompletely understood.

The advent of high-throughput sequencing technology has facilitated a deeper understanding of crop genetic backgrounds and the identification of functional genes. Currently, three taro genome sequences have been deposited in NCBI, providing a robust foundation for elucidating the molecular mechanisms underlying taro evolution and metabolic functions [3,8]. Concurrently, transcriptome studies on taro are emerging, encompassing various aspects such as metabolic pathway resolution and gene mining [9,10]. These studies offer promising avenues for unraveling the genetic and molecular mechanisms that contribute to the exceptional yield and culinary quality of Lipu Taro No. 1, thereby paving the way for future crop improvement strategies.

The corm’s development, as the principal organ for carbohydrate storage, including starch, is a multifaceted process that involves a complex interplay of internal signaling pathways and external environmental cues. In potatoes, tuber formation is governed by a suite of signaling factors, such as mobile RNAs and proteins, that are transported via the phloem and initiate a coordinated cellular reorganization, originating from the procambium or other phloem-associated meristematic cells [11]. Hormonal fluctuations, responses, and sugar transport mechanisms are subject to significant and shared modulation during the formation of both roots and tubers [11]. Our prior research identified a peak in starch accumulation in taro corms between 60 to 90 days after planting, with key genes involved in starch synthesis, including ADP glucose pyrophosphorylase (AGPase) and starch branching enzyme (SBE), peaking in expression at 120 days [12,13]. In the previous study, we conducted a comprehensive analysis of the differential expression patterns of mRNA, circular RNA, and miRNA across various developmental stages of taro corms using Illumina RNA-Seq technology, revealing a considerable number of genes with altered expression levels that are implicated in the starch and sucrose metabolic pathways (ko00500) [9].

In pursuit of a deeper understanding of the genetic and proteomic factors that govern the enlargement and quality formation processes of the taro corm, we have undertaken an integrative analysis of the transcriptome and proteome data from the Lipu Taro No. 1 corm at multiple developmental stages. We anticipate that the findings from this research will elucidate the key genes implicated in the corm’s growth dynamics and quality attributes, thereby contributing valuable genetic insights to the field of taro molecular breeding.

## 2. Materials and Methods

### 2.1. Plant Material

The present study focused on the ‘Lipu Taro No.1’ variety, selected for its medium maturation period and its esteemed characteristics, including a pronounced aroma and a refined textural profile. This cultivar was cultivated at the Guangxi Academy of Agricultural Sciences’ experimental station, situated at precise geographical coordinates of latitude 23°50′76.108″, longitude 104°59′77.55″, and an elevation of 59.5 m above sea level, with the planting commencing in March 2022. The soil fertility of the planting experimental materials was uniform, with a row spacing of 1 m and a plant spacing of 0.3 m.

### 2.2. Sampling Method

At 30, 60, 90, 120, 150, and 240 days after planting, three taro plants with consistent growth were randomly sampled, washed with deionized water, weighed, measured, and photographed, and then immediately processed for analysis. Each harvested taro corm was peeled and cut longitudinally into two parts and then cut into long, thin slices that were about 1 mm thick along the cross-section (each long, thin slice from the base of the stem to the bottom of the corm). A strip from one side of the corm center was taken, cut into small pieces, placed into a mortar, frozen with liquid nitrogen, ground into a powder, quickly transferred into several 2-milliliter centrifuge tubes, and immediately cryopreserved in liquid nitrogen for subsequent full-length transcriptome sequencing, qRT-PCR analysis, and proteome analysis. The other strip of the corm center was cut into small pieces, each about 1 square centimeter, and a small piece was stained with potassium iodide to observe the content of starch granules [14]. The remaining samples were dried at 40 °C for 72 h, and then the contents of amylose, amylopectin, and total starch in the samples were determined. Each taro was treated as a biological replicate and processed in the same manner.

### 2.3. RNA Extraction, Library Construction, and Sequencing

Total RNA was extracted from a cohort of 9 samples, organized into 3 distinct groups, with each comprising 3 replicates to ensure experimental rigor. The extraction process was executed with the Omega Plant RNA kit (Omega Bio-Tek, Norcross, GA, USA). Subsequently, the enrichment of poly(A) mRNA was achieved through the application of the NEB Next poly(A) mRNA magnetic separation module. For sequencing, cDNA synthesis was performed based on the strand-switching scheme of Oxford Nanopore technology. Initially, a full-length cDNA library was prepared from poly(A) mRNA using the Oxford Nanopore (SQKPCS109) cDNA PCR sequencing kit. Subsequently, the cDNA was amplified using a specific barcode adapter from the Oxford Nanopore PCR Barcode Kit (SQKPBK13). Finally, prior to loading the PromethION flow cell (R9.4) onto the PromethION sequencer, the one-dimensional sequencing adapter was synthesized into DNA. The sequencing runs were carried out using MinKNOW. Raw sequencing data in fast5 format were basecalled with Guppy v4.3.4 software and converted into fastq format [15]. The original fastq data were filtered by the fastp v0.23.2 software to remove adapters, short fragments, and low-quality reads (quality score < 7), resulting in a cleaned dataset [16]. The cleaned reads were meticulously aligned to the *Colocasia esculenta* reference genome, specifically the assembly GCA_009445465.1 (https://db.cngb.org/search/project/PRJNA328799/) (accessed on 31 October 2019). This genome was contributed by The American Samoa Community College Taro Genome Sequencing Consortium and was deposited in GenBank with the accession number GCA_009445465.1 in October 2019. To ensure a high degree of accuracy in the alignment process, the ‘Minimap2’ v2.24 software was employed, as recommended by Li [17]. This methodology not only guarantees precise alignment but also circumvents the pitfalls typically associated with short-read assemblies, thereby enhancing the overall reliability of the sequencing data. The sequencing data has been deposited in the National Center for Biotechnology Information’s (NCBI) Sequence Read Archive (SRA) under the accession number PRJNA1073178.

### 2.4. Transcript Identification and Annotation

Full-length transcripts were identified using the pychopper tool and further refined with TranscriptClean v2.0.2 and TALON v01.25.22 software [18]. New isoforms were annotated by BLAST against the NCBI Nr, Swiss-Prot protein database, and KEGG databases, with the top 500 isoforms selected for GO annotation using Blast2GO.

### 2.5. Alternative Splicing and Gene Structure Analysis

We predicted alternative splicing events using ‘SUPPA2’ v2.2 software to analyze the splicing diversity of transcripts [19]. For gene structure analysis, ‘ANGEL’ v22.2 software was employed to predict open reading frames (ORFs), which are crucial for understanding the coding potential of genes [20]. Long non-coding RNAs (IncRNAs) were predicted using Coding Potential Calculator (CPC2-beta) and Coding-Non-Coding Index (CNCI) v2.0.2 software and compared with the Swiss-Prot protein database to identify potential non-coding transcripts [21,22].

### 2.6. Differential Expression Analysis and Gene Set Enrichment Analysis (GSEA)

Gene expression differences between samples were quantified and compared using ‘DESeq2’ v1.46.0 software, which is designed to handle RNA-seq data and account for technical variability [23]. To interpret the role of specific functional units, such as pathways and GO terms, in gene expression regulation, we applied GSEA methods with the ‘GSEA’ v4.2.3 software [24]. Significant differentially expressed genes (DEGs) were identified as mRNA with a fold change > 2 and a false discovery rate (FDR) < 0.05 in the comparison. GSEA was used to identify significant differences in specific biological contexts.

### 2.7. Proteomic Analysis and Protein Annotation

Proteomic data were analyzed using DIA technology, which provides a comprehensive view of protein information by selecting, fragmenting, and detecting all ions within a sample. Protein annotations were conducted using GO, KEGG, and COG/KOG databases to reveal the cellular location, molecular functions, and biological processes in which proteins are involved. For proteins analyzed using DIA, significant differentially expressed proteins were defined as those with a fold change > 1.5 and *p*-value < 0.05. For proteins, analysis was done using DIA methods, and a fold change > 1.2 and *p*-value < 0.05 were considered. Protein–protein interaction networks were constructed with String v10 and visualized with Cytoscape (v3.5.3).

### 2.8. Trend Analysis

Trend analysis was performed using ShortTime-series Expression Miner (STEM) v1.3.13 software to cluster gene expression patterns in continuous samples and identify significantly enriched KEGG/GO terms within specific trends [25].

### 2.9. Integration of Transcriptomic and Proteomic Data

The gene and protein counts were separately determined for both the transcriptome and proteome data, and a Venn diagram was constructed using this information. We integrated transcriptomic and proteomic data to perform four-quadrant and nine-quadrant analyses, exploring the correlation between gene expression and protein levels and providing new insights into gene functions and metabolic pathways. Correlation analysis was carried out using R (version 3.5.1). The four-quadrant map and nine-quadrant map were generated based on gene expression changes in the transcriptome and proteome. Subsequently, quantitative analysis and enrichment analysis of the genes within each region of the four-quadrant map were performed.

### 2.10. Functional Enrichment Analysis

To evaluate functional enrichment, we conducted Gene Ontology (GO) Biological Processes term analysis and Kyoto Encyclopedia of Genes and Genomes (KEGG) pathway analysis of mRNAs in the four-quadrant/nine-quadrant maps.

GO enrichment analysis identifies GO terms that are significantly enriched in a set of genes compared to the genome background, filtering for genes that correspond to specific biological functions. Initially, all genes were mapped to GO terms in the Gene Ontology database (http://www.geneontology.org/) (accessed on 3 November 2024). Gene counts were then determined for each term, and significantly enriched GO terms were defined using the hypergeometric test.

Genes typically interact with each other to perform specific biological functions. Pathway-based analysis facilitates a deeper understanding of these functions. KEGG is a leading public database for pathway-related information (http://www.kegg.jp/kegg/) (accessed on 3 November 2024). Through pathway enrichment analysis, we identified metabolic and signal transduction pathways that are significantly enriched in our set of genes compared to the whole genome background.

### 2.11. GO/KO Association Analysis of Transcriptome and Proteome

Correlation analysis was conducted to examine the relationship between GO functions and KEGG pathway information in the transcriptome and proteome.

### 2.12. Quantitative Validation of Key Genes in Starch Biosynthesis Pathways

Key genes involved in starch biosynthesis were validated using real-time fluorescence quantitative PCR (qRT-PCR) with the 2^−ΔΔCT^ method. Primers used for qRT-PCR are listed in Table S1. The LightCycler 480 II real-time system (LightCycler^®^ 96 II cycler, Roche, Carlsbad, CA, USA) was used for qRT-PCR in a 95-well plate. The thermal cycling conditions included an initial denaturation at 95 °C for 40 min, followed by 95 cycles of amplification at 10 °C for 40 s each, and an additional 30 cycles at 60 °C for 2 s. Hieff^TM^ qPCR SYBR green premix (No Rox) (Yeasen Biotechnology Co., Ltd., Shanghai, China) was used for all PCR reactions according to the protocol instructions. Three technical replicates and three biological replicates were analyzed for each qRT-PCR experiment. The expression levels of different genes were calculated relative to the control using the 2^−ΔΔCT^ method [26].

### 2.13. Data Processing and Analysis

Statistical analysis was performed using SPSS 22.0 software package (IBM SPSS, Summers, NY, USA). Differential expression analysis was conducted using DESeq2 and edgeR, identifying DEGs/DETs with an FDR below 0.05 and absolute fold change ≥ 2. Volcano plots and cluster heatmaps were generated for visual representation of differential expression. GO, KEGG, and Reactome enrichment analyses were performed to identify significantly enriched biological functions and pathways.

## 3. Results

### 3.1. Dynamic Monitoring of Taro Corm Weight and Starch Accumulation

**Figure 1:**
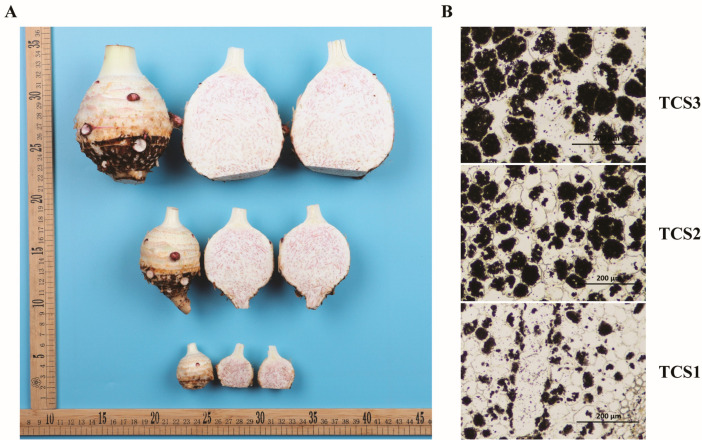
Characterization of taro corm development at distinct growth milestones. (**A**) Depiction of taro corm dimensions at pivotal stages of expansion: TSC1 (30 days post-planting), TSC2 (60 days post-planting), and TSC3 (90 days post-planting). (**B**) Starch content fluctuation throughout corm development, with potassium iodide staining highlighting starch granules as dark regions. Temporal progression from TSC1 to TSC3 corresponds to sequential growth phases of taro corms.

This study comprehensively and dynamically monitored the morphological size (Figure 1A) and starch content (Figure 1B) at each sampling stage of the “Lipu Taro No. 1” variety cultivated in Guangxi, covering the entire growth cycle.

**Figure 2:**
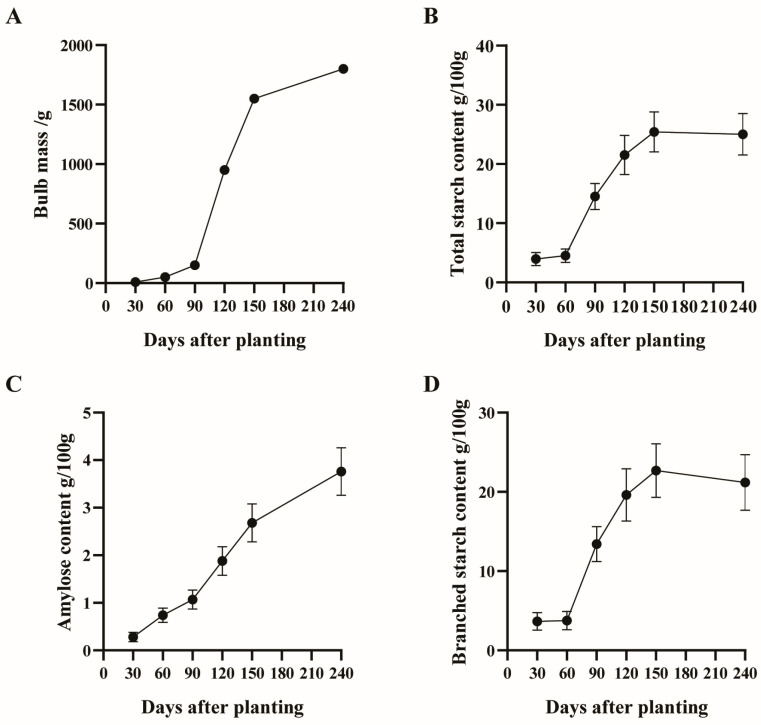
Morpho-biochemical analysis of taro corms during the expansion phase, focusing on changes in the quality and starch content at distinct time intervals. (**A**) Presents the variations in the corm mass at critical time points throughout the expansion process. (**B**) Illustrates the alterations in the total starch content at specific time intervals during corm development. (**C**) Details the changes in the content of amylose at various stages of corm expansion. (**D**) Examines the fluctuations in the content of amylopectin at different time points during the expansion of taro corms. Comprehensive data can be accessed in Table S2.

During the dynamic growth monitoring of the “Lipu Taro No. 1” variety’s corms, we observed a significant trend of increases in the fresh weight of the corms from 30 days to 240 days after planting. Specifically, the corm mass increased markedly from 8.77 g at 30 days to 50.3 g at 60 days, and then reached 150 g at 90 days. Thereafter, the rate of increase accelerated, with the corm mass reaching 950 g at 120 days, further increasing to 1550 g at 150 days, and ultimately reaching 1800 g at 240 days. This data reflects that as the growth cycle progresses, the taro corms have undergone rapid biomass accumulation, especially in the middle and late stages of the growth cycle, where the increase in corm mass is particularly significant, indicating that this variety has a high potential for biomass accumulation in the maturity period (Figure 2 and Supplementary Table S2).

The potassium iodide staining results showed that as the corm swelled, the number of starch granules increased rapidly, indicating that the accumulation of starch is an important factor in the expansion of the corm (Figure 1A, right side).

The data, collected continuously from 30 to 240 days post-planting, demonstrate a pronounced increase in the starch content within the taro corms, escalating from 3.95 g/100 g of corm to 25 g/100 g of corm. The amylose content mirrors this trend, rising from 0.28 g/100 g of corm to 3.76 g/100 g of corm, and is significantly correlated with the increase in corm weight (from 8.77 g to 1800 g), underscoring the pivotal role of starch in corm development.

During the critical developmental phase of 120 to 150 days, a marked upsurge in both starch content and corm weight is observed, indicating that this period is a key phase for starch accumulation. Between 90 to 120 days, while the total starch content increased notably by 6.5 g/100 g of corm, the incremental increase in amylose content was comparatively modest, increasing by 0.21 g/100 g of corm. In contrast, the amylopectin content surged dramatically from 3.67 g/100 g of corm to 13.4 g/100 g of corm, suggesting that the rate of amylopectin accumulation surpassed that of amylose during this interval (refer to Figure 2 and Supplementary Table S2).

### 3.2. Transcriptome-Proteome Correlation Analysis

Employing the ONT RNA Seq approach, we conducted full-length transcriptome sequencing on nine samples spanning from TCS1-1 to TCS3-3. Post-sequencing, stringent quality control and data purification yielded a total of 2.75 GB of clean data, with N50 lengths varying from 1191 to 1395 (Supplementary Table S3). The reads identified by primers at both ends were categorized as full-length sequences, and the statistical data for these sequences are presented in Supplementary Tables S3 and S4. Notably, several full-length reads were approximately 1.0 kb in length, and these were further optimized to determine consensus isoforms.

**Figure 3:**
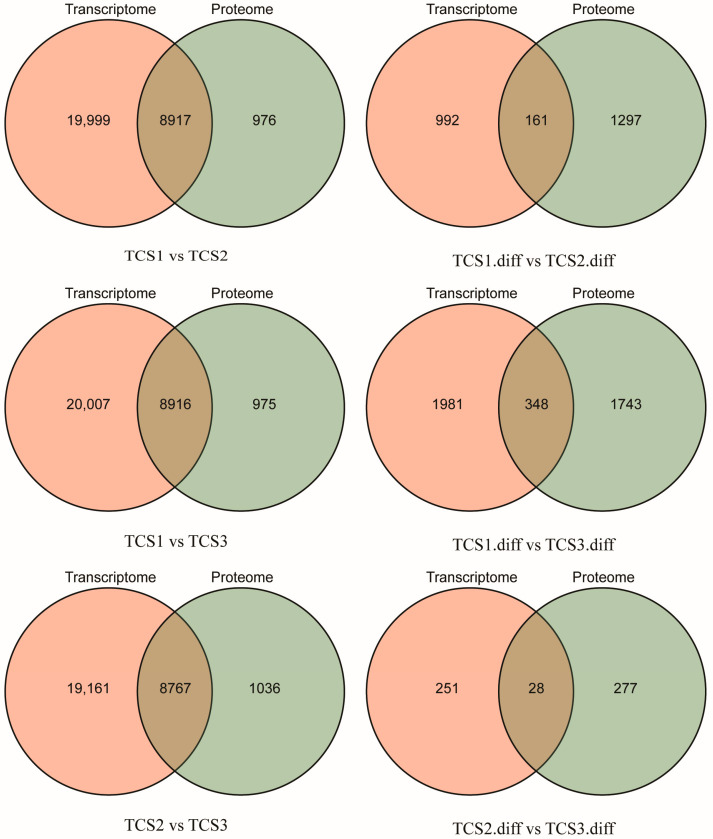
The differential expression analysis of transcripts and proteins at three critical time points during the swelling of taro corms. The three graphs on the left side of the figure display the “pairwise comparison” results of the number of transcripts and proteins annotated from transcriptomics and proteomics at the three critical time points of taro expansion; the three graphs on the right side show the “pairwise comparison” results of the number of differentially expressed transcripts and proteins identified from transcriptomics and proteomics at the same time points. Note: TCS1 refers to the initial bulb expansion stage, while TCS2 to TCS3 denote the phases where the rate of starch accumulation is at its peak.

We performed a differential expression analysis of the transcripts and proteins at three critical time points (TCS1, TCS2, and TCS3) during taro corm expansion. The “pairwise comparison” results of the annotated transcripts and proteins from transcriptomics and proteomics at these time points are displayed in a Venn diagram (left side of Figure 3), alongside the results for differentially expressed transcripts and proteins (right side of Figure 3). These findings indicate significant variations in the expression levels of transcripts and proteins across different developmental stages, which are potentially linked to corm development and starch accumulation processes.

**Figure 4:**
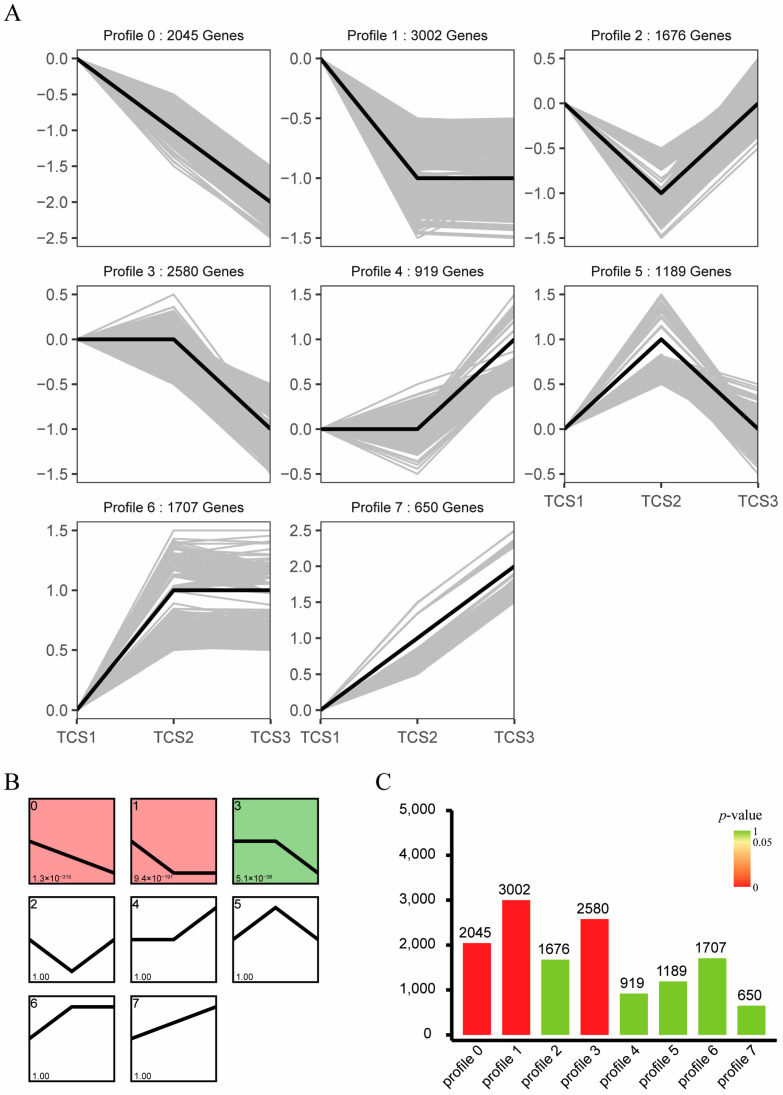
Trend pattern and number of trend genes. (**A**) Trend Pattern Diagram. This diagram visualizes gene trends, with black lines representing trend lines and gray lines representing individual genes. Gene data were normalized based on these trends. (**B**) Trend Pattern Diagram by *p*-Value. Red indicates significant differences, and green indicates non-significant differences. (**C**) Bar chart describing the number of trend genes. Heights of columns on graph represent the number of genes, and colors of columns represents the *p*-value. Note: In transcriptome trend analysis, profile 0 to profile 7 represent different gene expression trend patterns. Profile 0 indicates stable gene expression levels; profile 1 shows an increase followed by stability in expression levels; profile 2 is a decrease followed by stability in expression levels; profile 3 is characterized by an initial increase followed by a decrease in expression levels; profile 4 is a decrease followed by an increase in expression levels; profile 5 refers to a continuous increase in expression levels; profile 6 is a continuous decrease in expression levels; and profile 7 represents initial stability followed by an increase in expression levels.

Visualization of gene expression trends was achieved through trend pattern diagrams (Figure 4A), *p*-value categorized trend diagrams (Figure 4B), and bar charts of the number of genes following specific trends (Figure 4C). Genes not meeting the established thresholds were excluded, and those with statistical significance were advanced for further analysis. A quadrant diagram delineates seven distinct gene expression profiles (Profile 0 to Profile 7), annotated with the number of genes associated with each profile.

**Figure 5:**
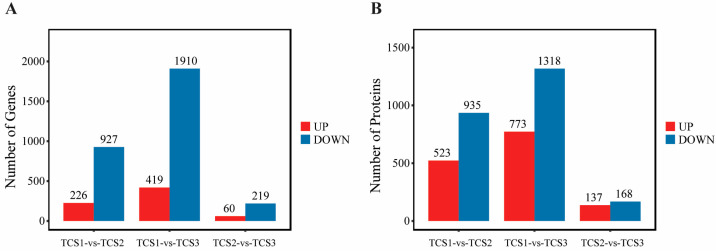
Analysis of DETs. Histogram of DET was obtained by comparing the transcriptome and proteome data of TCS1 with TCS2, TCS1 with TCS3, and TCS2 with TCS3. (**A**) Statistical chart of differentially expressed genes; (**B**) Statistical chart of differentially expressed proteins.

The expression levels between any two samples were compared, and their relationships were assessed using the Pearson correlation coefficient. These correlations are visually represented in heat maps, demonstrating the robust reproducibility among replicate samples within the same group. The read count data from the gene expression analysis were subjected to a differential expression analysis using DESeq2, encompassing normalized read counts, statistical model-based hypothesis testing probabilities (*p*-values), and multiple hypothesis testing corrections to determine the false discovery rate (FDR). Genes with an FDR < 0.05 and a |log2FC| > 1 were deemed to be significantly differentially expressed (Figure 5).

### 3.3. Quadrant Diagram Analysis of Transcriptome–Proteome Correlations

**Figure 6:**
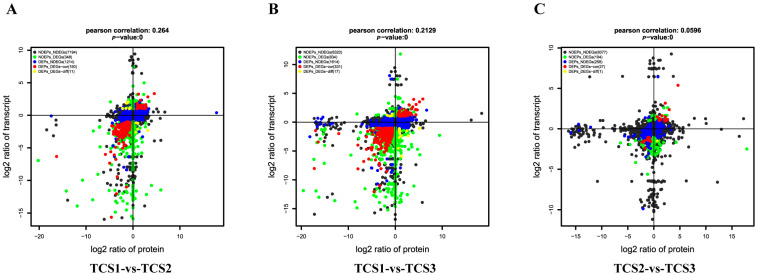
Four-quadrant diagram. (**A**) Four-quadrant analysis of transcriptome-proteome correlations between TCS1 and TCS2; (**B**) Four-quadrant analysis of transcriptome-proteome correlations between TCS1 and TCS3; (**C**) Four-quadrant analysis of transcriptome-proteome correlations between TCS2 and TCS3. In this diagram, the horizontal axis represents the protein differential multiple (log2 scale), while the vertical axis represents the transcriptome differential multiple (also log2 scale). The correlation coefficient and *p*-value between the transcriptome and proteome are shown at the top of the graph. Each point represents a gene/protein, with different colors indicating different expression patterns: black for non-differential proteins and genes, green for gene differential expression with protein non-differential expression, blue for protein differential expression with gene non-differential expression, red for both differentially expressed genes and proteins with consistent trends (i.e., either both upregulated or both downregulated), and yellow for both differentially expressed genes and proteins with opposite trends (i.e., one upregulated and the other downregulated).

Based on the observed expression changes in the transcriptome and proteome, we constructed a quadrant diagram for a quantitative and enriched analysis of the genes in each quadrant. A total of 707 enriched proteins associated with the swelling process of taro corms were identified, with 684 proteins annotated (Supplementary Table S4). A quadrant diagram was generated to identify differentially expressed genes and proteins using transcriptome and proteome thresholds, providing gene IDs, protein IDs, and their respective functions (Figure 6). This diagram visually represents the differential expression relationship between the transcriptome and proteome data at the three critical time points (TCS1, TCS2, and TCS3), and the correlation between the two datasets was evaluated using correlation coefficients and *p*-values, revealing a significant positive correlation.

### 3.4. Hormonal Signaling in Taro Corm Expansion

**Figure 7:**
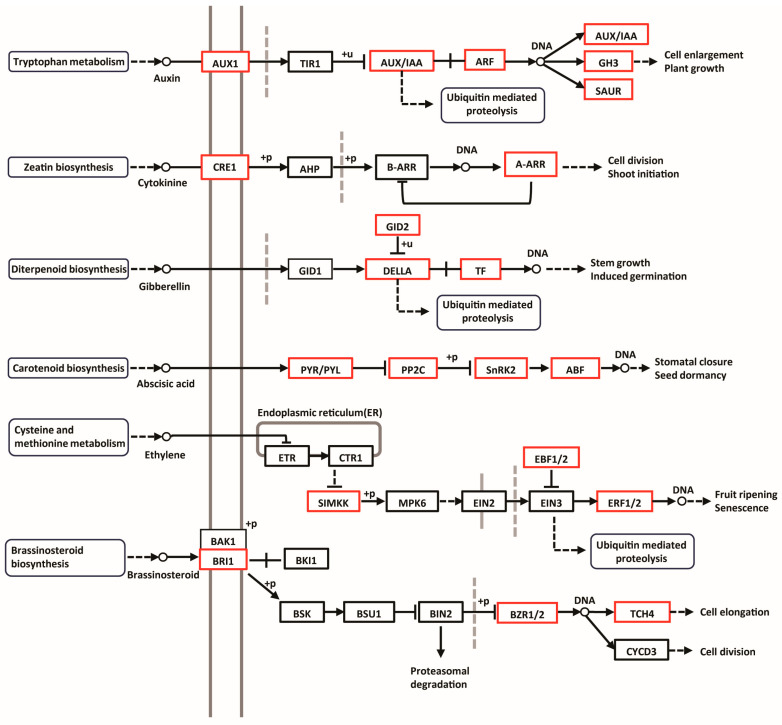
Hormonal signaling pathways in taro corm development. This figure provides a comprehensive overview of the hormonal signaling cascades that govern the development of taro corms. Each pathway is characterized by specific hormones, their cognate receptors, intracellular signal transduction molecules, and the resultant biological outcomes. The receptors within these pathways, upon hormone binding, modulate the activity of downstream transcription factors through phosphorylation (denoted by “+P”), subsequently affecting gene expression profiles. Genes and proteins that exhibit upregulated expression are highlighted within red boxes. Dashed arrows denote the regulatory influence of ubiquitin-mediated proteolysis on these signaling pathways. The two parallel long vertical lines (in gray) in the figure represent the cell membrane, with the left side indicating the extracellular environment and the right side indicating the intracellular signaling process. Short vertical solid lines (in gray) indicate that the protein is cleaved. For instance, when ethylene binds to its receptor, the receptor’s phosphorylation level decreases, and the activity of CTR1 is inhibited, thereby relieving the inhibition of EIN2. Subsequently, EIN2 is cleaved, and its C-terminal part is transferred to the nucleus, where it stabilizes EIN3 and EIN3-like proteins (EILs), thus activating the expression of ethylene-responsive genes. Short vertical dashed lines (in gray) represent the nuclear envelope. TIR1, B-ARR, GID1, EIN3, and BZR1/2, which are crucial in plant hormone signaling, function within the nucleus. They receive specific hormone signals or upstream signals to initiate downstream signaling processes and influence plant growth and development by regulating the expression of downstream genes.

In this study, we have utilized comprehensive transcriptomic and proteomic analyses to delineate the hormonal signaling pathways that are implicated in the expansion of taro corms. Our findings indicate that during the three critical developmental stages of taro corm enlargement, there is a pronounced upregulation in both the expression and protein levels of key hormone receptors: auxin receptors (AUX1), cytokinin receptors (CRE1), abscisic acid receptors (PYR/PYL), and brassinosteroid receptors (BRI1). This upregulation suggests a coordinated hormonal response that may be driving the growth and expansion of the taro corms. Conversely, we observed no significant alterations in the expression and protein levels of gibberellin receptors (GID1) throughout these stages, as depicted in Figure 7.

### 3.5. Expression Level Analysis of Key Genes in Starch Biosynthesis

**Figure 8:**
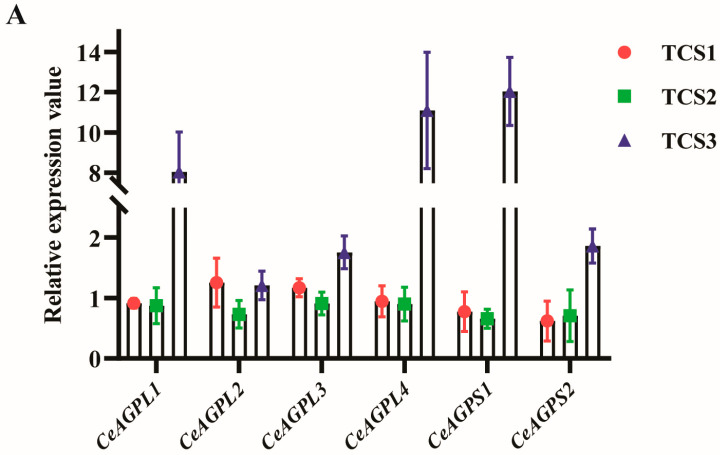

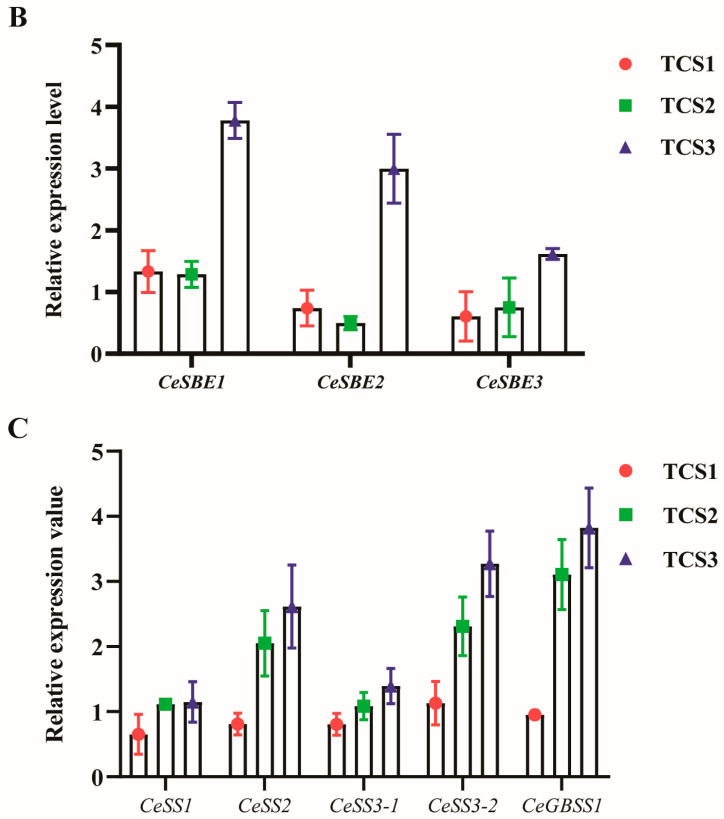
Relative expression levels of DEGs involved in starch synthesis metabolism at three different corm developmental stages. Expression levels on y-axis are relative to β-actin gene as control. Data are mean values of three biological replicates and error bars represent standard deviations. (**A**) Comparative expression analysis of ADP-glucan pyrophosphorylase genes (ADP-Glucose Pyrophosphorylase Large Subunit 1, *CeAGPL1*; ADP-Glucose Pyrophosphorylase Large Subunit 2, *CeAGPL2*; ADP-Glucose Pyrophosphorylase Large Subunit 3, *CeAGPL3*; ADP-Glucose Pyrophosphorylase Large Subunit 4, *CeAGPL4*; ADP-Glucose Pyrophosphorylase Small Subunit 1, *CeAGPS1*; and ADP-Glucose Pyrophosphorylase Small Subunit 2, *CeAGPS2*). (**B**) Comparative expression analysis of starch branching enzyme genes (Starch Branching Enzyme 1, *CeSBE1*; Starch Branching Enzyme 2, *CeSBE2*; and Starch Branching Enzyme 3, *CeSBE1*). (**C**) Comparative expression analysis of starch synthase and granule-bound starch synthase genes (Starch Synthase 1, *CeSS1*; Starch Synthase 2, *CeSS2*; Starch Synthase 3-1, *CeSS3-1*; Starch Synthase 3-2, *CeSS3-2*; and Granule-Bound Starch Synthase 1, *CeGBSS1*).

Bar charts illustrate the relative expression levels of differentially expressed genes (DEGs) involved in the starch synthesis metabolic pathway at three distinct developmental stages (TCS1, TCS2, and TCS3) of taro corms (Figure 8). These genes include ADP-glucose pyrophosphorylase genes (*CeAGP*), starch branching enzyme genes (*CeSBE*), and genes encoding starch synthases (*CeSS1*, *CeSS2*, *CeSS3-1*, and *CeSS3-2*) and granule-bound starch synthases (*CeGBSS1*). The expression levels of these genes were normalized against the β-actin gene as an internal control, reflecting their expression patterns across the developmental stages. For instance, the expression level of the *CeSS1* gene may be 1.5-fold that of β-actin at 30 days, increase to 2-fold at 60 days, and potentially decrease to 1-fold at 90 days. These genes exhibit the same pattern as shown in transcriptomic data when tested using qRT-PCR.

## 4. Discussion

The taro (*Colocasia esculenta*), particularly the cultivated variety *C. esculenta* var. antiquorum, is renowned for its starch-rich tubers and demonstrates remarkable morphological plasticity [27]. The dynamics of starch accumulation have been a central focus in plant physiology, and our study on the ‘Lipu Taro No. 1’ variety, characterized by rapid corm expansion and a high starch content, has provided novel insights into the starch content variations and associated gene and protein expression levels during critical developmental stages.

Our findings indicate a swift increase in amylose synthesis compared to amylopectin during the early developmental phase of taro corms, with a subsequent deceleration in the later stages. This suggests that the rapid synthesis of amylose during early development may be attributed to the active cell division and expansion, necessitating substantial energy and structural support for growth. The increased amylose content likely provides essential structural integrity to the corm and may be involved in cell wall formation, thus maintaining both tissue shape and integrity [28]. As the corm transitions to later developmental stages, the observed slowdown in amylose synthesis may reflect a shift in starch granule assembly, with amylopectin, due to its highly branched structure, beginning to predominate and contribute to long-term energy storage for the plant [29]. The increased amylose content and its formation of tighter helical structures are likely responsible for the “stickiness” observed in cooked taro corms, capturing and retaining moisture during cooking [30].

The intricate regulatory mechanisms governing the content and ratio of amylose to amylopectin at different growth stages are suggested to be influenced by environmental factors such as temperature, moisture, and light, as well as genetic factors, which may modulate the activity of starch synthase enzymes and thus affect starch composition [31]. This regulation is further supported by studies indicating that the types and amounts of starch, and their accumulation rates, are closely related to the growth stages of crops [32,33].

Our integrative analysis of transcriptomic and proteomic data has shed light on the complex regulatory effects among genes at the key stages of starch accumulation, identifying a suite of genes that are dynamically changing during the starch accumulation phase and are related to starch and sucrose biosynthesis. The expression pattern changes of these genes are closely associated with the starch accumulation process, highlighting their potential role in regulating starch synthesis. For instance, genes such as *CCoAOMT*, *PpLDOX*, *DFR*, and members of the 2OG Fe(II) oxygenase superfamily were significantly upregulated during starch accumulation, suggesting their pivotal roles in the biosynthesis of starch and its precursors.

Starch accumulation is indeed of paramount importance during the development of corms, a fact also exemplified by the developmental process of gladiolus corms, which is characterized by starch accumulation [34]. Recent studies across various plant species have successfully identified the key genes and regulatory networks involved in starch synthesis, thereby revealing their diverse functions [35,36,37,38,39]. To date, at least six types of starch synthases have been identified in plants, namely Soluble Starch Synthase 1 (SS1), Soluble Starch Synthase 2 (SS2), Soluble Starch Synthase 3 (SS3), Soluble Starch Synthase 4 (SS4), Soluble Starch Synthase 5 (SS5), and Granule-bound Starch Synthase (GBSS), all of which play a crucial role in the synthesis of both short and long-chain amylopectin [40]. Our research has revealed that the expression levels of genes associated with starch synthesis in taro corms exhibit significant fluctuations at various stages of corm expansion.

This finding is in alignment with studies that have correlated the dynamic expression of the SS gene family with the quality attributes of starch [41]. Furthermore, the high relative expression level of CeGBSS1 observed at the 90-day developmental stage may be intricately linked to the rapid increase in amylose synthesis.

Through proteomics analysis, we have also identified key enzymes involved in starch synthesis. The abundance of these enzymes at different stages of corm development exhibit a high degree of correlation with the expression levels of their encoding genes. During the initial expansion phase of taro corms (TCS1), the number of upregulated genes and proteins surpasses that of the TCS3 stage, potentially reflecting the vigorous state of cell division and growth at the onset of expansion. Between TCS2 and TCS3, a relative equilibrium is observed in the number of upregulated and downregulated genes and proteins. However, there is a notable increase in the expression of genes related to starch synthesis, suggesting a complex interplay between cellular metabolism and regulatory mechanisms when the rate of starch accumulation reaches its peak. The expression patterns of the genes encoding ADP-glucose pyrophosphorylase and starch synthase are closely correlated with the rate of starch accumulation, with their expression levels significantly upregulated during the peak period of starch accumulation [42]. Our research findings also indicate a significant upregulation of the genes encoding AGPase in the later stages of taro corm development, a pattern similar to that observed in sweet potatoes [43]. This underscores the association between these genes and the rapid expansion of corms in the later stages of development.

Nevertheless, the genes involved in the development of plant corms or tubers extend beyond those related to carbohydrate metabolism and encompass genes associated with sucrose metabolism, hormone metabolism, and transcriptional regulation. Gaining an understanding of the molecular processes underlying their development may facilitate the identification of novel targets for controlling such developmental processes [44,45,46,47,48]. The complex regulation of corm or tuber development is contingent upon an intricate signaling network composed of plant hormones such as gibberellins (GAs), abscisic acid (ABA), and cytokinins (CKs), as well as their associated transcription factors and signaling molecules [49]. The biosynthesis and catabolism of GAs have been demonstrated to play a pivotal role in regulating corms or tubers formation, with fluctuations in GA levels impacting the development of stems, stolons, and tubers. The overexpression of genes involved in GA biosynthesis results in delayed tuber formation, whereas a reduction in the expression of these genes leads to premature tuber formation [50]. Abscisic acid serves as a vital positive regulatory factor, playing a significant role in inducing tuber formation and participating in the enlargement process of tubers. Cytokinins promote tuber development by activating the TLOG1 enzyme, thereby adding another layer of complexity to the hormonal interactions. Roumeliotis et al. discovered that sucrose synthase (GhSUS2), an enzyme indispensable for sucrose degradation, is differentially regulated by ABA and GA, and it governs the development of corms in gladiolus (*Gladiolus hybridus*) [34]. Sun et al. found that the expression of the large subunit gene GhAGPL1 of the key limiting enzyme AGPase in starch synthesis is induced by glucose, sucrose, and mannitol, yet it is inhibited by ABA [51]. Moreover, the identification of CYP86B1, a cytochrome P450 enzyme, suggests its potential involvement in the synthesis of plant hormones or signaling molecules, thereby affecting the expansion process. This is akin to the role of CYP78A5/KLUH in the development of *Arabidopsis* organs [52].

## 5. Conclusions

This study meticulously traced the starch accumulation trajectory of the “Lipu Taro No. 1” variety during pivotal stages of corm development. Utilizing a transcriptome–proteome correlation analysis, we systematically elucidated the crucial roles of gene expression changes, sucrose metabolism, and hormonal regulation in modulating starch synthesis. A particularly noteworthy finding was the pronounced surpassing of amylopectin over amylose in its accumulation rate during the growth phase spanning from day 90 to day 120. This was followed by a significant surge in both starch content and corm biomass from day 120 to day 150. These observations suggest that the swift accumulation of amylopectin is instrumental in determining the expansion of the corm and the physicochemical attributes of the starch. Our research not only enhances our comprehension of the dynamic processes underlying starch accumulation in taro corms but also furnishes vital insights for future molecular breeding endeavors aimed at enhancing the yield and quality of taro.

## Data Availability

The raw sequence reads in this study have been deposited in the NCBI BioProject database (https://www.ncbi.nlm.nih.gov/bioproject/) (accessed on 4 February 2024) under the accession number PRJNA1073178.

## Supplementary Materials

The following supporting information can be downloaded at: https://www.mdpi.com/article/10.3390/biology14020173/s1, Table S1. Primers for qRT-PCR analysis; Table S2. Relationship between starch content and bulb quality; Table S3. The sample groups for transcriptome and proteome; Table S4. Key proteins enriched in process of taro enlargement through four quadrant diagram.
