# Supplementary material for: Integrated Transcriptome and Proteome Analysis Provides New Insights into Starch and Sucrose Metabolism and Regulation of Corm Expansion Process in Colocasia esculenta
Source: Biology (Basel). 2025 Feb 8;14(2):173. doi: 10.3390/biology14020173 (PMC11851817; doi:10.3390/biology14020173)
Supplement: Supplementary file 1 [file biology-14-00173-s001.zip › Table S1.pdf]

Table S1. The qPCR primers used in this study.

Gene name	Forward primer sequence (5'-3')	Reverse primer sequence (5'-3')
Actin	TCTGGCACCACACCTTCTAC	Actin-GACACACCGTCACCAGAGTC
CeAGPL1	TGGCAGGCTTATCTCTTCAGG	TTGGTAGGTGGCAAGTAACGA
CeAGPL2	TCGTCGTCCTCCTCCTCATC	CGGCGGTTCAAGTGTCATAGA
CeAGPL3	GGCTGATATTACCATTGCTTGTCT	CAGTTGTATCCACCTCCATTGC
CeAGPL4	CTACACATCGCCTCGTCACTT	CAACACCGCAATGATCCAAGAA
CeAGPS1	GGAACTGCTGATGCTGTGAG	TCTCGGTGTGCTTGAATGAAC
CeAGPS2	GCATCAACAGCAACATCACCAA	AACCTCCACGAATCCATCCTTG
CeSBE1	GCATTAGGAGGCGAAGGCTAT	CCACTGACGGCGACACTTAT
CeSBE2	CGGAAGCACGCACCTTATGA	GCTGGACAGGCTGAAGTAACA
CeSBE3	ACACCATTCGGTCACCATTCC	CTGCCTTGCTCACCTCTTCC
CeSS1	CCTCGGACCAGCACATACAAT	GGCAATCAGGACGAACATCAAG
CeSS2	CGACCTGAAGTTCCTGTTGTTG	GCATATCCTGACTGACCATCCA
CeSS3-1	TGCGATATGGCTCTATACCTGT	GCTCCAGACCTTGTGCTTGA
CeSS3-2	GGCACATAGAACCTGGAGCAT	ACCATCAGCATACCACCAATCA
CeSS4	CCAGAGCCATCCGCACATT	GGCACAGAACCATAACGCATT
CeGBSS1	AGTCTGCTATGTCTTGCTGCTT	AGAACTCCAGTGTGCCAATCAT
