# Supplementary material for: Integrated Transcriptome and Proteome Analysis Provides New Insights into Starch and Sucrose Metabolism and Regulation of Corm Expansion Process in Colocasia esculenta
Source: Biology (Basel). 2025 Feb 8;14(2):173. doi: 10.3390/biology14020173 (PMC11851817; doi:10.3390/biology14020173)
Supplement: Supplementary file 1 [file biology-14-00173-s001.zip › Table S2.pdf]

Table S2. Relationship between Starch Content and Bulb Quality.

Days after planting	Total starch content g/100g	Amylose content g/100g	Amylopectin content g/100g	Bulb mass /g
30d	3.95	0.28	3.67	8.77
60d	4.51	0.74	3.77	50.3
90d	14.5	1.07	13.4	150
120d	21.5	1.88	19.6	950
150d	25.4	2.68	22.7	1550
240d	25	3.76	21.2	1800
