# Supplementary material for: Integrated Transcriptome and Proteome Analysis Provides New Insights into Starch and Sucrose Metabolism and Regulation of Corm Expansion Process in Colocasia esculenta
Source: Biology (Basel). 2025 Feb 8;14(2):173. doi: 10.3390/biology14020173 (PMC11851817; doi:10.3390/biology14020173)
Supplement: Supplementary file 1 [file biology-14-00173-s001.zip › Table S3.pdf]

Table S3. The sample groups for transcriptome and proteome.

Experimental material	TCS1	TCS2	TCS3
Days after planting	30	60	90
Mass of taro bulbs/g	8.77	50.3	150
Grouping	Low starch content	Moderate starch content	High starch content
Total starch content g/100g	3.95	4.51	14.5
amylose content g/100g	0.28	0.74	1.07
branched starch content g/100g	3.67	3.77	13.4
